# Psychometric Properties of the Teleprimary Care Oral Health Clinical Information System (TPC-OHCIS) Questionnaire Using the Rasch Model

**DOI:** 10.7759/cureus.63064

**Published:** 2024-06-24

**Authors:** Shahida Ismail, Rosnah Sutan, Roszita Ibrahim, Fairuz Zana Mohd Rathi

**Affiliations:** 1 Department of Public Health Medicine, Universiti Kebangsaan Malaysia, Kuala Lumpur, MYS; 2 Division of Family Health Development, Ministry of Health Malaysia, Putrajaya, MYS

**Keywords:** rasch model, tpc-ohcis, dhis, telehealth, psychometric testing, reliability, psychometric properties

## Abstract

Background

The Tele Primary Care Oral Health Clinical Information System (TPC-OHCIS) was implemented in Malaysia to digitalize health care and reduce numerous ground-level manual tasks. This study measures the psychometric properties of the TPC-OHCIS questionnaire among healthcare workers (HCWs) at primary healthcare clinics (PHC).

Method

A pilot study was conducted at PHC, which implemented the TPC-OHCIS application for service delivery. The questionnaire contained 65 items with four response categories, grouped into four scales: technology, organization, external support, and human resource. The questionnaire items were analyzed using the Rasch model in Winsteps 3.72.3.

Results

There were 319 respondents who participated (98.8%). The Cronbach alpha was 0.93. The construct validity was determined by a positive point measure correlation (PMC) value, with an infit and outfit mean square (MNSQ) range of 0.4-1.5 and a Z-standardized (ZSTD) range of -2.0 to 2.0. The person and item reliability were 0.93 and 0.97, respectively, indicating excellent reliability. The questionnaire was unidimensional, where the raw variance explained by measures was >40%.

Conclusion

The questionnaire was deemed fit for an actual survey after 18 items had been deleted. It has good psychometric properties and is practically applicable for evaluating HCWs on the TPC-OHCIS application implementation process monitoring using the local Malay language. High reliability and unidimensionality were achieved, supporting its use in digital healthcare. With this validated questionnaire, it will enhance digital healthcare implementation and streamline manual tasks.

## Introduction

Telehealth is defined as revolutionizing the delivery of healthcare services by using information and communication technologies to exchange valid information for the diagnosis, treatment, and prevention of diseases [1,2]. The telehealth application allows healthcare workers (HCWs) to share data between facilities regardless of distance. The telehealth data-sharing platform offers various features, including electronic medical records (EMR), online appointments, electronic prescriptions, public health surveillance, vaccination, environmental health, institutional health management, and online medical education among HCWs [1,2]. Globally, telehealth systems have improved resource coordination from different localities, facilitating access to essential healthcare services in the community [3,4]. In 1991, Canada became the first country to use telehealth, subsequently adopted by the United Kingdom, Hong Kong, Taiwan, Korea, Jordan, Saudi Arabia, Singapore, and 70 other nations utilizing a District Health Information System (DHIS) [5,6]. Telehealth has been adapted and renamed in various countries while retaining the same function of integrating information technology into healthcare. For instance, it is known as Opth Web in Singapore, HAKEEM in Jordan, and Tele Primary Care Oral Health Clinical Information System (TPC-OHCIS) in Malaysia, among others [5]. In developed countries, telehealth applications are more commonly used in conventional healthcare, whereas in developing countries, they serve as an alternative to conventional healthcare. However, the requirements for these applications are more demanding in developing countries, as they need to provide broad populations with basic healthcare services and bridge the distance between rural areas and specialized hospitals typically located in large cities [7,8].

In Malaysia, the TPC-OHCIS application is a form of telehealth initiated to enhance the continuity of care through a specific digital system designed for primary care service delivery, covering a 'womb to tomb' approach [9,10]. The TPC-OHCIS application was pilot-tested at the Seremban Health Clinic in Negeri Sembilan and was designed to support service delivery in the outpatient department, maternal and child health unit (MCH), dental healthcare outpatient, pharmacy, medical laboratory, radiography, and disease surveillance monitoring. Later, the application was expanded to 122 other clinics in Malaysia [10]. TPC-OHCIS is a web-based application that allows HCWs to record data during service provision at the clinic or during home visits, as it can function offline [10]. The system automatically updates record data upon internet availability. The TPC-OHCIS application is free of physical boundaries, allowing patients to walk into any governmental healthcare facility equipped with this system and receive treatment as their data are accessible at any of these locations [9].

Since the COVID-19 outbreak in February 2020, Malaysia has experienced various health service delivery disruptions at primary care levels [11]. Consequently, there have been significant interruptions in the MCH services and school health programs due to school closures following the imposed movement restriction order to curb COVID-19 infection [5]. Fortunately, the availability of digital technologies such as TPC-OHCIS has enhanced healthcare delivery despite the movement restrictions [9]. To date, there has been no evaluation of the TPC-OHCIS application’s usage on improving the efficiency of health indicator monitoring. Therefore, this study aims to assess the validity and reliability of the TPC-OHCIS questionnaire for the implementation of the application in primary healthcare settings during the COVID-19 pandemic. The findings of this study will be used to provide insights for better TPC-OHCIS implementation and utilization in healthcare delivery in Malaysia. Assessing the ability of the healthcare delivery system to adapt and shift towards providing services through telehealth applications is important to avoid catastrophic consequences.

## Materials and methods

Study design and sample

A cross-sectional study was conducted at primary healthcare clinics (PHCs) equipped with the TPC-OHCIS application in three selected states: Federal Territory of Kuala Lumpur and Putrajaya, Selangor, and Pahang. The TPC-OHCIS questionnaire was developed based on various combinations of the technology-organization-environment theory [12], human organization technology-fit theory (HOT-fit) [13], diffusion of innovation (DOI) theory [14], and technology acceptance model theory [15]. A total of 319 respondents were recruited through convenience sampling, meeting the inclusion criteria of having used the TPC-OHCIS application in handling MCH services at the PHC for at least one month. The targeted respondents were MCH staff directly involved in data input, management, and interpretation using the TPC-OHCIS application. For validation of the TPC-OHCIS questionnaire, a convenience sampling technique was used in three of the fourteen states of Malaysia. Later, the validated questionnaire will be used to measure national TPC-OHCIS implementation across all states. The validation process was conducted during the COVID-19 pandemic, and only available HCWs who met the inclusion criteria were invited to participate in the study.

Instrument

The questionnaire was developed using a standard tool for managing health information for decision-making, employing the Technology‐Organization‐Environment (TOE) framework [8], and adapted to local service needs in compliance with the four aforementioned theories [12-15]. Ten experts, including the primary health program manager at the TPC-OHCIS Unit of the Ministry of Health Malaysia (MOH), researchers, and the Chief of Information Technology, were requested to review the 65-item TPC-OHCIS questionnaire. The relevance of each questionnaire item was rated on a scale of one to three (1 = not clear, 2 = revision needed, 3 = very clear) for clarity, and the essentiality of the questionnaire was determined by a similar scale (1 = not essential, 2 = useful but not essential, 3 = essential). The questionnaire was made bilingual in English and Bahasa Malaysia after a forward-backward translation conducted by two linguistics experts. Three experts in related fields conducted a content validity evaluation. The expert panel scores were analyzed with an acceptable cut-off content validity index score of 1 [16]. Once reviewed, the forward-backward translation was repeated. Subsequently, a pilot study was conducted with respondents including medical doctors and nurses at the PHC equipped with the TPC-OHCIS application and responsible for the MCH units. Reliability analysis was performed using the Rasch model in Winsteps 3.72.3 for Cronbach’s alpha and Cohen’s kappa testing [17,18].

Four scales were determined in the questionnaire: technology, organization, environment, and human resources (Appendix 1). The technology scale included 17 items and four subscales: relative advantage (ARA1-5), compatibility (ACOM6-9), complexity (ACOMPLEX10-13), and security concern (ASEC14-17). The organization scale comprised 18 items and four subscales: presence of a champion (BCHAMP18-22), infrastructure (BINFRA23-27), top management support (BTP28-31), and financial resources (BFIN32-35). The environment scale contained only four items (CVEN36-39). Lastly, the human resource scale included 26 items and six subscales: Staff competency in information technology systems (DPT 40-44), knowledge of the TPC-OHCIS (DEIS 45-50), clinical information technology competency (DCIT 51-54), perceived innovativeness of information technology officers (DCIO 55-57), perceived ease of use (DPEU 58-61), and perceived usefulness (DPU 62-65).

Data analysis

Each questionnaire item was analyzed using the Rasch model in Winsteps 3.72.3. The instrument's reliability and validity were evaluated in a 5-step process using the program. Initially, the person and item polarities were examined. Problematic objects and individuals were identified using fit statistics and were either eliminated or altered. Next, the person and item separation (s) indexes and other reliability metrics were assessed. Subsequently, the unidimensionality and local independence of the inventory were calculated. The item difficulty level was examined using the item-person map (Wright map).

Person and Item Polarity

All items and dimensions measured were required to have positive point measure correlation (PMC) values [17]. The PMC should be >0.30, which demonstrates that the items can measure the proposed dimensions effectively [17,18]. An item that demonstrates high polarity can differentiate among respondents [19]. Items and people with negative PMC values were deleted as they were considered outliers (person) and indicated that a dimension was not being measured (item) [20].

Person and Item Fit

The ability of the items to measure the latent trait or dimension was determined by analyzing the item fit [17-21]. The person fit was examined to identify individuals who may have contributed an abnormal reaction compared to the norm [17]. The range 0.4-1.5 for the mean square (MNSQ) outfit and infit was deemed appropriate for the person and item fit of polytomous data (Likert scale). The Z-standardized (ZSTD) value was between -2 and +2.

Person and Item Reliability

As the inventory had a high Cronbach alpha (Kuder-Richardson 20 formula KR-20) score, all items within the same dimension demonstrated outstanding internal consistency [17,19]. According to the Rasch model, a value ≥ 0.80 indicates high reliability for both the item and the person [17]. The Rasch model has high personal reliability if the items can distinguish among respondents for the variables or constructs being measured [17,18,22].

Person and Item Separation Indexes

The person separation index is defined as the ability of the questions to distinguish and categorize respondents' qualities or characteristics from the measuring variables [20]. The item separation index is used to confirm item hierarchy, distributing items based on item difficulty [23]. Person and item separation indexes with higher values indicate excellent separation [24]. Values in the 1.5-2.0 range are considered appropriate, whereas good person and item separation requires a value > 3.0.

Unidimensional and Local Independence

Unidimensionality and local independence ensure items contribute to a single construct [17,24]. Item correlation is defined when all items relate to the same variable [17,18]. A minimum of 40% of the raw variance is required for an accurate measurement of unidimensionality [17,19]. Unexplained variance in the first contrast should not exceed 15% [17,19]. The eigenvalue of the first contrast should fall within the 1.4-3.0 range [17]. Local dependence is identified by correlations between items [19]. Standard residual correlation is conducted between items to determine dependency, where a value > 0.70 indicates that the items should be deleted as they share the same traits and are highly dependent on each other [17,18,20].

Item-Person Map (Wright Map)

Wright maps demonstrate interval-level measurement by logit scale to identify the hierarchy of a respondent’s ability based on item difficulty [17]. The difficulty-based item distribution enables the evaluation of how well the questions (items) are formulated [17]. The best Wright maps fit when both item and person scores are zero (0) values at the same level, which demonstrates that the items are well answered by the respondents with an almost identical understanding of the items [17]. Unfortunately, a Wright map is seldom perfect. The gap between the items, respondents’ measure, respondents’ fit, item fit, and item measure encourages further examination of whether the model fits the formulated theory [25].

Research ethic

The Research Ethical Committee of the National University of Malaysia and the National Medical Research Registry granted ethical approval for the conduct of this study. We adhered to the ethical principles to protect the dignity, rights, and welfare of the respondents. Research information sheets on the research description, purpose, benefit, and personal data confidentiality were provided before the respondent consented to sign the consent form to participate in the study.

## Results

A total of 319 respondents were analyzed in this pilot study, which was conducted at PHC with the TPC-OHCIS application in three states of Malaysia: Federal Territory of Kuala Lumpur-Putrajaya, Selangor, and Pahang. Demographic data (age, gender, race) and job-related information (designation, length of service, and role in the MCH unit as a TPC-OHCIS user, vendor, or information officer) were collected for the respondents. Four respondents were identified as upper outliers and six as lower outliers; these were removed from the data set. Items DPTC40, CVEN39, ASEC14, and CVEN38, which appeared at the bottom of the Wright map and were considered outliers (more than 2 standard deviations), were also deleted from the questionnaire. Ultimately, only 309 respondents and 61 items were used for further analysis.

Item fits

Table 1 describes the overall item analysis by presenting the summary statistics for the initial dimension. There were four scales (dimensions): Technology (ARA 1-5, ACOMPATIBLE6-9, ACOMPLEX10-13, ASEC14-17), Organization (BCHAMP18-22, BINFRA23-26, BTP27-31, BFIN32-35), Environment (CVEN36-39), and Human Resource (DPTC40-44, DEISK 45-50, DCITEX 51-54, DCIO 55-57, DPEU 58-65). Table 1 demonstrates individual item mean square infit and outfit (MNSQ) estimates, standardized fit infit and outfit (ZSTD) values, and the PMC coefficient. The PMC should yield a positive value, as a negative value indicates that the questionnaire did not measure the intended parameter [13,16]. Items ACOMPATIBLE8, ACOMPATIBLE9, ACOMPLEX10, ASEC14, ASEC15, BTP31, DEISK46, DEISK47, DEISK48, DCITEX51, DCIO56, and DCIO57, which yielded negative PMC values, were therefore deleted. The same items also had MNSQ values greater than 1.5, where the normal range for the MNSQ outfit is between 0.4 and 1.5 [17].

**Table 1:** Descriptive analysis of all items of the TPC-OHCIS questionnaire. TPC-OHCIS: Tele primary care oral health clinical information system; MEASURE: Item Difficulty Estimate; MNSQ: Mean Square; ZSTD: Standardized Fit.

Dimension	Item	MEASURE	Model standard error	Infit		Outfit		Point measure correlation coefficient
				MNSQ	ZSTD	MNSQ	ZSTD	
Technology	Using the TPC-OHCIS application enables me to do my work quickly (ARA1)	0.49	0.08	0.87	-1.7	0.85	-2.0	0.74
	Using the TPC-OHCIS application improves my quality of work. (ARA2)	0.54	0.08	0.84	-2.2	0.81	-2.6	0.71
	Using the TPC-OHCIS application enhances my job effectiveness. (ARA3)	0.48	0.08	0.97	-0.4	0.94	-0.8	0.70
	Using the TPC-OHCIS application increases my productivity. (ARA4)	0.07	0.08	0.88	-1.7	0.87	-1.7	0.71
	Using the TPC-OHCIS application makes my job easier (ARA5)	0.20	0.08	0.67	-4.9	0.67	-4.9	0.75
	TPC-OHCIS application can be easily accessed across multiple platforms (ACOMPATIBLE6)	0.30	0.08	0.74	-3.8	0.73	-3.8	0.76
	TPC-OHCIS application user interfaces provide transparent access to all platforms (ACOMPATIBLE7)	0.31	0.08	0.85	-2.1	0.84	-2.2	0.71
	Data received from other devices (tablet /laptop/smartphone) outside health facilities in the TPC-OHCIS application can be easily merged into the database for analysis (ACOMPATIBLE8)	-0.84	0.08	1.84	9.6	1.92	9.9	-0.21
	Information data is shared seamlessly across our organization regardless of the location (ACOMPATIBLE9)	-0.78	0.08	1.72	8.4	1.78	9.0	-0.21
	I do not know enough about the TPC-OHCIS application to handle my job satisfactorily. (ACOMPLEX10)	-0.99	0.08	1.84	9.6	1.93	9.9	-0.21
	I need a long time to understand and familiar with the TPC-OHCIS application (ACOMPLEX11)	-1.15	0.09	1.72	7.6	1.78	8.1	0.15
	I do not find enough time to study and upgrade my technology skills before using the TPC-OHCIS application. (ACOMPLEX12)	0.32	0.08	1.02	0.4	1.04	0.5	0.55
	I often find it too complex to understand and use this TPC-OHCIS application (ACOMPLEX13)	0.46	0.08	0.83	-2.3	0.83	-2.3	0.60
	I feel secure in using the TPC-OHCIS application, keying patients’ data, and sharing it across my organization (ASEC14)	-1.08	0.08	1.85	9.7	1.96	9.9	-0.29
	I would feel safe using the TPC-OHCIS application to retrieve patient data (ASEC15)	-0.89	0.08	1.32	4.2	1.38	4.9	-0.07
	I am concerned about data patient leakage. (ASEC16)	0.45	0.08	0.78	-3.0	0.80	-2.8	0.62
	I am concerned about how much I can trust the vendor (ASEC17)	0.73	0.08	0.72	-3.9	0.72	-4.1	0.67
Organization	A specified liaison officer will inform top managers and vendors about the faulty TPC-OHCIS application (BCHAMP18)	0.54	0.08	0.63	-5.6	0.62	-5.8	0.69
	A specified liaison officer plays a role in upgrading the TPC-OHCIS application for users’ needs. (BCHAMP19)	0.57	0.08	0.64	-5.3	0.64	-5.5	0.74
	A specified liaison officer has a good relationship with both vendors and top managers (BCHAMP20)	0.16	0.08	1.00	0.0	1.00	0.0	0.53
	A specified liaison officer can train staff to use the TPC-OHCIS application well. (BCHAMP21)	-0.19	0.08	1.46	5.5	1.46	5.5	0.38
	A specified liaison officer provides training/courses for the users a few times a year. (BCHAMP22)	-0.42	0.08	1.02	0.4	1.03	0.4	0.54
	We have enough computers for the TPC-OHCIS application use. (BINFRA23)	0.07	0.08	0.67	-5.0	0.66	-5.2	0.61
	We have a reliable computer network in our use. (BINFRA24)	0.36	0.08	0.76	-3.4	0.75	-3.6	0.60
	Appropriate hardware, software, and network infrastructures were in place before the TPC-OHCIS application implementation (BINFRA25)	0.81	0.08	0.79	-2.9	0.76	-3.4	0.63
	Presence of integrated Information System applications encompassing different functional areas (BINFRA26)	0.48	0.08	0.71	-4.2	0.70	-4.3	0.70
	Top Management always supports and encourages using the TPC-OHCIS application for job-related tasks (BTP27)	0.81	-2.7	0.80	-2.7	0.80	-2.7	0.59
	Top management provides the necessary help and resources to enable staff to use the TPC-OHCIS application (BTP28)	0.26	0.08	0.63	-5.6	0.63	-5.7	0.67
	Top management provides good access to hardware when staff need it. (BTP29)	0.29	0.08	0.56	-6.8	0.57	-6.7	0.72
	Top management gives feedback to vendors on every dismay or unsatisfactory comment from staff (BTP30)	-0.24	0.08	0.83	-2.4	0.84	-2.4	0.58
	Top management provides good access to the TPC-OHCIS application when staff needs it. (BTP31)	-0.44	0.08	1.61	7.2	1.69	7.9	-0.11
	The organization provides sufficient financial aid to coordinate the implementation of TPC-OHCIS (BFIN32).	-0.24	0.08	1.30	3.8	1.31	3.9	0.34
	I find it difficult to use the TPC-OHCIS application because it cannot be upgraded due to insufficient budget. (BFIN33)	-0.79	0.08	1.07	1.0	1.12	1.7	0.32
	Enough computers are available to access the TPC-OHCIS application. (BFIN34)	0.02	0.08	0.62	-5.9	0.62	-5.8	0.65
	We easily get obsolete computer replacements. (BFIN35)	0.06	0.08	0.57	-6.8	0.57	-6.8	0.67
Environment	The vendor entertains each one of our complaints dutifully (CVEN36)	-0.19	0.08	0.59	-6.5	0.59	-6.4	0.59
	The vendor can upgrade the TPC-OHCIS application according to our needs. (CVEN37)	-0.20	0.08	0.72	-4.1	0.73	-4.0	0.58
	The system vendor attended the technical meetings quite frequently. (CVEN38)	-1.98	0.09	1.89	8.8	2.64	9.9	0.14
	I have a platform to voice problems regarding the TPC-OHCIS application directly to the vendors (CVEN39)	-1.84	0.08	1.74	7.8	2.21	9.9	0.12
Human resource	I don’t know how to use a computer (DPTC40)	-1.42	0.08	1.62	7.4	1.75	8.3	0.04
	I never used to work online (DPTC41)	0.37	0.08	0.92	-1.1	0.92	-1.0	0.63
	I need people’s help to use a computer (DPTC42)	0.29	0.08	0.88	-1.6	0.88	-1.6	0.67
	I like to work using the online system (DPTC43)	0.03	0.08	1.04	0.6	1.04	0.6	0.44
	The TPC-OHCIS application is easy to operate (DPTC44)	-.05	0.08	0.92	-1.0	0.94	-0.8	0.45
	I have enough training/courses before working with the TPC-OHCIS application (DEISK45)	0.11	0.08	0.80	-2.8	0.82	-2.6	0.66
	It took me only a few days to master the TPC-OHCIS application well. (DEISK46)	-0.98	0.08	2.23	9.9	2.38	9.9	-0.39
	The TPC-OHCIS application facilitates task management (DEISK47)	-0.58	0.08	1.93	9.9	1.99	9.9	-0.19
	The TPC-OHCIS application is hard to use (DEISK48)	-0.54	0.08	1.89	9.9	1.95	9.9	-0.18
	I have to open many interfaces to key in one patient’s data (DEISK49)	0.49	0.08	0.80	-2.8	0.81	-2.7	0.50
	TPC-OHCIS application takes time because I have to open so many interfaces (DEISK50)	-0.34	0.08	0.87	-1.8	0.90	-1.5	0.30
	I have confidence in my ability to operate the TPC-OHCIS application. (DCITEX51)	-0.53	0.08	1.18	2.4	1.23	3.0	-0.01
	I have the expertise regarding Information technology to provide valuable knowledge to the organization (DCITEX52)	0.13	0.08	0.92	-1.0	0.96	-0.5	0.32
	It doesn’t make any difference whether I add/ share knowledge with others related to the usage of the TPC-OHCIS application. (DCITEX53)	0.15	0.08	0.57	-6.7	0.58	-6.6	0.60
	Other employees can provide more valuable knowledge about the application’s use. (DCITEX54)	0.15	0.08	0.52	-7.7	0.53	-7.5	0.66
	The Information Technology Officer is actively considering the introduction of new technology to solve to organization’s problem (DCIO55)	-0.06	0.08	0.66	-5.2	0.67	-5.0	0.45
	The Information Technology Officer tries to keep a technological leading edge by adopting new technology early (DCIO56)	-0.96	0.08	1.64	7.7	1.70	8.3	-0.17
	The Information Technology Officer tends to take risks in the decision-making of new technology introduction (DCIO57)	-0.89	0.08	1.98	9.9	2.12	9.9	-0.42
	I often become confused every time I use the TPC-OHCIS application (DPEU58)	0.37	0.08	0.75	-3.6	0.74	-3.7	0.70
	Interacting with the TPC-OHCIS application is frequently frustrating (DPEU59)	0.23	0.08	0.61	-6.0	0.61	-5.9	0.73
	I find the TPC-OHCIS application makes my job easier (DPEU60)	0.04	0.08	1.01	0.2	1.04	0.6	0.49
	The TPC-OHCIS application provides useful guidance in performing tasks (DPEU61)	0.46	0.08	0.67	-4.9	0.68	-4.7	0.70
	My job would be hard to perform without the TPC-OHCIS application (DPU62)	0.33	0.08	0.84	-2.3	0.84	-2.2	0.67
	Using the TPC-OHCIS application improves my job performance (DPU63)	0.24	0.08	0.66	-5.1	0.66	-5.0	0.68
	Using the TPC-OHCIS application saves me job time (DPU64)	0.33	0.08	1.00	0.0	1.00	0.1	0.55
	Using the TPC-OHCIS application supports critical aspects of my job (DPU65)	0.24	0.08	0.79	-3.0	0.79	-3.0	0.59
Mean (SD)		0.00 (0.49)	0.08 (0.00)	1.00 (0.43)	-0.7 (5.1)	1.02 (0.46)	-0.6 (5.2)	-

Reliability and separation indexes

The item and person reliability of the questionnaire were 0.97 and 0.90, respectively, which exceeded 0.90 and indicated high reliability and extreme acceptability [17,18]. The high person reliability demonstrated that the respondents in this pilot study exhibited varying abilities to respond to the questionnaire, which also relates to the findings from the Wright map [26]. The Cronbach's alpha (KR20) of 0.93 indicated good internal consistency and qualified it for subsequent actual data collection [17]. The high item reliability confirmed that the items could be accurately located according to the latent variable. In this study, the person separation index of 3.05 demonstrated that the 61 items could separate the respondents into three categories (agree, neither agree nor disagree, or disagree) regarding whether the TPC-OHCIS application made their jobs easier during the COVID-19 pandemic. A person separation index value greater than 3.0 is considered excellent separation [17].

Table 2 shows that the final round of analysis reflected that the results were satisfactory at both the person and item levels. Several indicators illustrate this improvement, as follows: (1) good person and item separation (3.53 and 6.14, respectively), and (2) acceptable person and item reliability (0.93 and 0.97, respectively).

**Table 2:** Item and person summary statistics for the modified TPC-OHCIS subscale. MEASURE: Item Calibration Estimated; MNSQ: Mean Square; ZSTD: Standardized Fit; RMSE: Root Mean Square Error. Real (Person level): RMSE: 0.20; True SD: 0.62; Item Separation: 3.05; Item Reliability: 0.90 Model (Person level): RMSE: 0.18; True SD: 0.62; Item Separation: 3.53; Item Reliability: 0.93 Real (Item level): RMSE: 0.08; True SD: 0.48; Item Separation: 5.70; Item Reliability: 0.97 Model (Item level): RMSE: 0.08; True SD: 0.48; Item Separation: 6.14; Item Reliability: 0.97

Statistic	Total score	Measure	Model error	Infit		Outfit	
				MNSQ	ZSTD	MNSQ	ZSTD
Person level (309 persons)							
Mean	141.00	-0.29	0.18	1.03	-0.80	1.02	-0.80
SD	21.30	0.65	0.01	0.72	4.10	0.74	4.10
Max	189.00	1.13	0.21	4.64	9.90	5.35	9.90
Min	98.00	-1.75	0.17	0.12	-9.60	0.11	-9.10
Item level (65 items)							
Mean	714.30	0.00	0.08	1.00	-0.70	1.02	-0.60
SD	82.10	0.49	0.00	0.43	5.10	0.46	5.20
Max	899.00	0.81	0.08	2.23	9.90	2.38	9.90
Min	586.00	-1.08	0.08	0.52	-7.70	0.53	-7.50

Unidimensional and local independence

**Figure 1:**
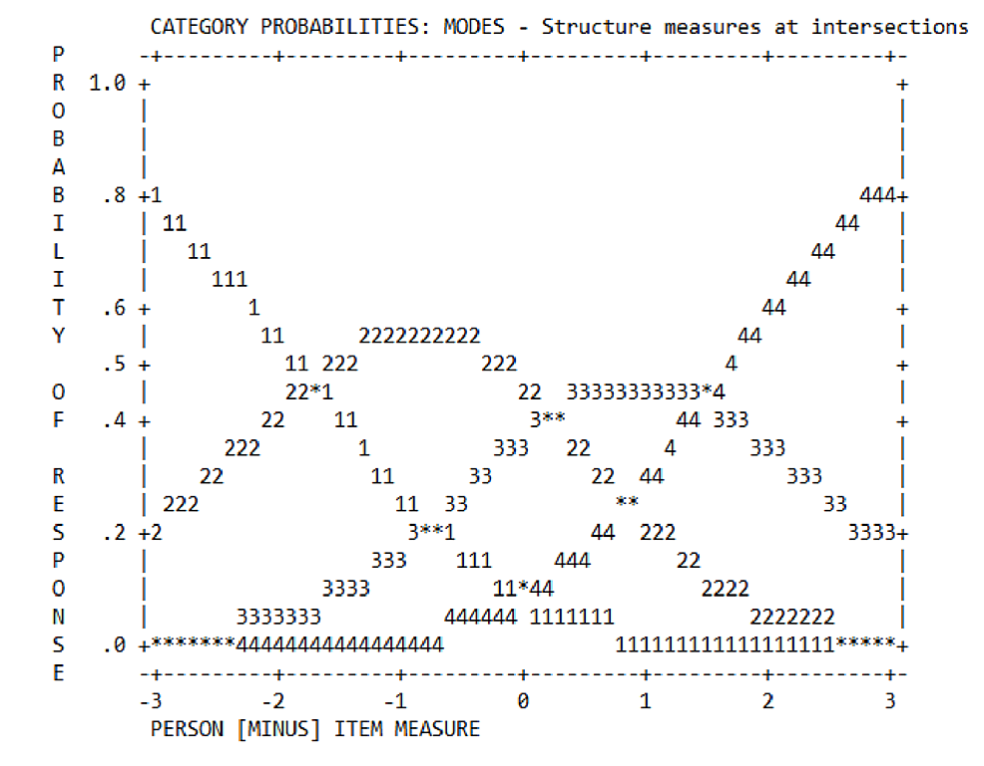
Format analysis for category probability curves for TPC-OHCIS items. 1: Strongly disagree; 2: Disagree; 3: Agree; 4: Strongly agree. TPC-OHCIS: Tele Primary Care Oral Health Clinical Information System.

Table 3 depicts the unidimensional analysis results based on the standardized residual variance. The table demonstrated that the raw variance explained by the measures was 40.5% (minimum requirement: 40%) and the model raw variance explained by the measures was 41%. This indicated that the construct validation was almost equivalent to the Rasch-predicted model. The Rasch model requires a minimum value of 40% for raw variance explained by measures and 60% for excellent unidimensionality [26]. Unexplained variance in the first contrast value should not exceed 15% for ideal measurement [22]. In this study, both the raw variances explained by measures (40.5%) and unexplained variance in the first contrast (8.7%) were achieved. In Rasch analysis, local dependence is defined to identify any correlations between the items [17]. Table 4 shows the standardized residual correlations used to identify dependent items. Items DEISK47, ARA1, ACOMPATIBLE9, and DPEU61 were deleted due to factor loadings greater than 0.7 (Figure 1). A higher standard residual correlation value greater than 0.70 indicated that these items were dependent and shared the same traits [14]. Items ARA2 and ARA3 were rephrased. Aside from the deleted items, all other items were considered valid for use in an actual study.

**Table 3:** Analysis of unidimensionality by the standardized residual variance (in eigenvalue units).

Residual variance	Empirical			Modeled
	-	%	%	%
Total raw variance in observations	82.4	100.0	-	100.0
Raw variance explained by measures	33.4	40.5	-	41.0
Raw variance explained by persons	17.6	21.3	-	21.5
Raw variance explained by items	15.8	19.2	-	19.4
Raw unexplained variance (total)	49.0	59.5	100.0	59.0
Unexplained variance in 1^st^ contrast	7.1	8.7	14.6	-
Unexplained variance in 2^nd ^contrast	4.3	5.2	8.7	-
Unexplained variance in 3^rd ^contrast	3.2	3.9	6.6	-
Unexplained variance in 4^th^ contrast	2.5	3.0	5.1	-
Unexplained variance in 5^th^ contrast	2.3	2.8	4.7	-

**Table 4:** Standardized residual correlations used to identify the dependent item.

Correlation	Entry number	Item	Entry number	Item
0.87	43	DEISK47	44	DEISK48
0.83	1	ARA1	2	ARA2
0.78	1	ARA1	3	ARA3
0.75	2	ARA2	3	ARA3
0.71	9	ACOMPATIBLE9	11	ACOMPLEX11
0.71	57	DPEU61	58	DPU62
0.70	10	ACOMPLEX10	11	ACOMPLEX11
0,67	9	ACOMPATIBLE9	10	ACOMPLEX10
0.67	8	ACOMPATIBLE8	9	ACOMPATIBLE9
0.67	20	BCHAMP21	31	BFIN32

Format analysis

Format analysis defines the scoring adequacy. In this pilot study, a 1-4 Likert scale was used. Table 5 demonstrates that the separation complied with the range of 1.4 < s < 5, which was the study's aim [17,26].

**Table 5:** Separation of scale.

Scale	Calculation	Comply/not
4-3	2.75 - 0.92 = 1.83	Comply
3–2	0.92 - (-0.83) = 1.75	Comply
2–1	-0.83 - (-2.90) = 2.07	Comply

Table 6 demonstrates that the respondents were able to differentiate and apply the scale as intended. The observed count in each category was significant (i.e., >10), showing a monotonic increase in category measures without any disorder. An increase in the distance between the threshold across categories aligns with Linacre’s liberal criteria [18]. However, the distances between the Andrich thresholds of adjacent categories were small (d < 1.4), presenting a problematic issue. The criteria are explained as follows: “a category represents too narrow a segment of the latent variable or corresponds to a concept that is poorly defined in the minds of the respondents.” The category probability curves illustrated smooth rolling hills (Figure 1).

**Table 6:** Category frequency and threshold values for the TPC-OHCIS dimensions. TPC-OHCIS: Tele Primary Care Oral Health Clinical Information System.

Category label	Observed count	%	Observed average	Sample expect	Infit Mean Square	Outfit Mean Square	Andrich Threshold	Category measure
1	3070	16	-0.99	-0.90	1.02	1.07	None	(-2.90)
2	8544	45	-0.46	-0.46	0.92	0.93	-1.71	-0.83
3	5527	29	0.21	0.05	0.73	0.70	0.23	0.92
4	1708	9	0.23	0.57	1.28	1.28	1.48	(2.75)

Person-item map

**Figure 2:**
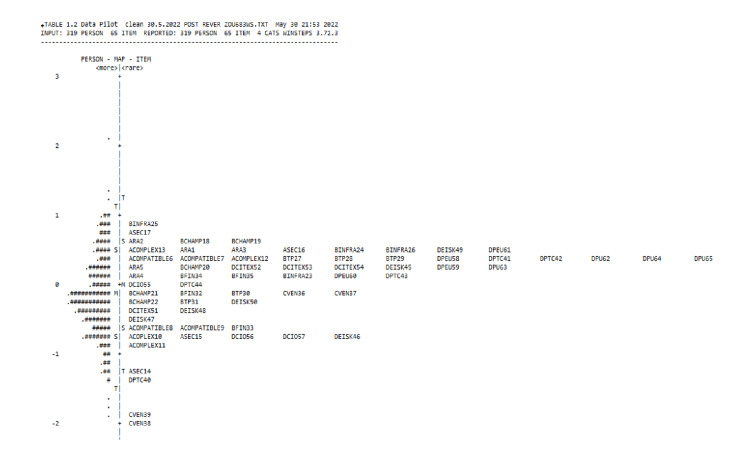
Wright item-person map for the TPC-OHCIS subscale. TPC-OHCIS: Tele Primary Care Oral Health Clinical Information System.

The Wright map illustrates the complexity of the items based on the logit scale [17]. In this study, the means of the item and person were similar (Figure 2), indicating that 50% of the respondents agreed with the questions, and 50% did not. Up to 68% of the respondents answering the questions were within 1 SD, enabling them to discern the true meaning of the items.

**Figure 3:**
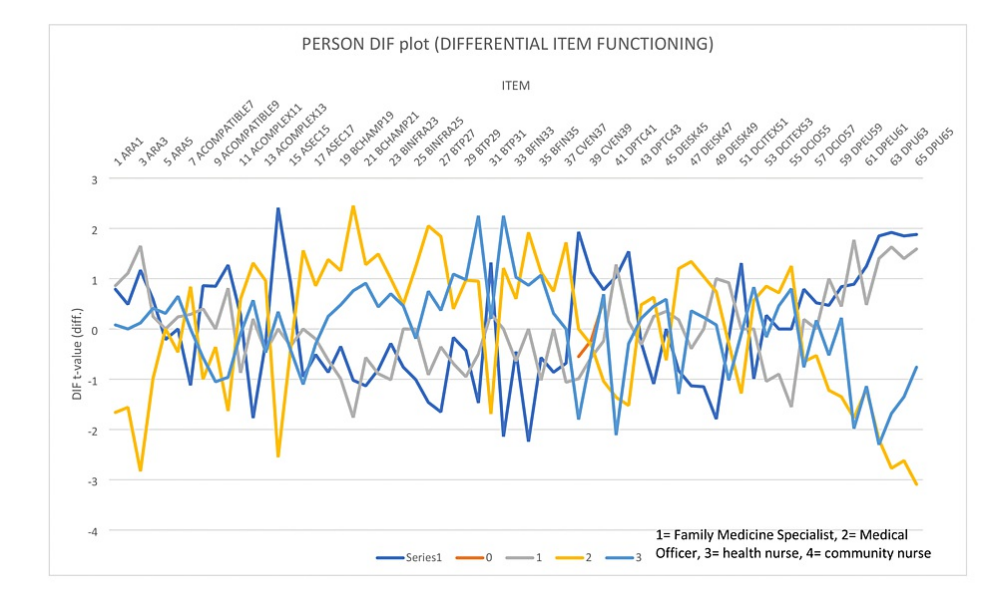
Person differential item functioning (DIF) plot according to respondent job category. 1: Family medicne; 2: Medical officer; 3: Staff nurse; 4: Community nurse.

Figure 3 shows the person differential item functioning (DIF), which indicates different probabilities of endorsing a given item on a multi-item scale after controlling for overall scale scores.

Pooled confirmatory factor analysis (CFA)

**Figure 4:**
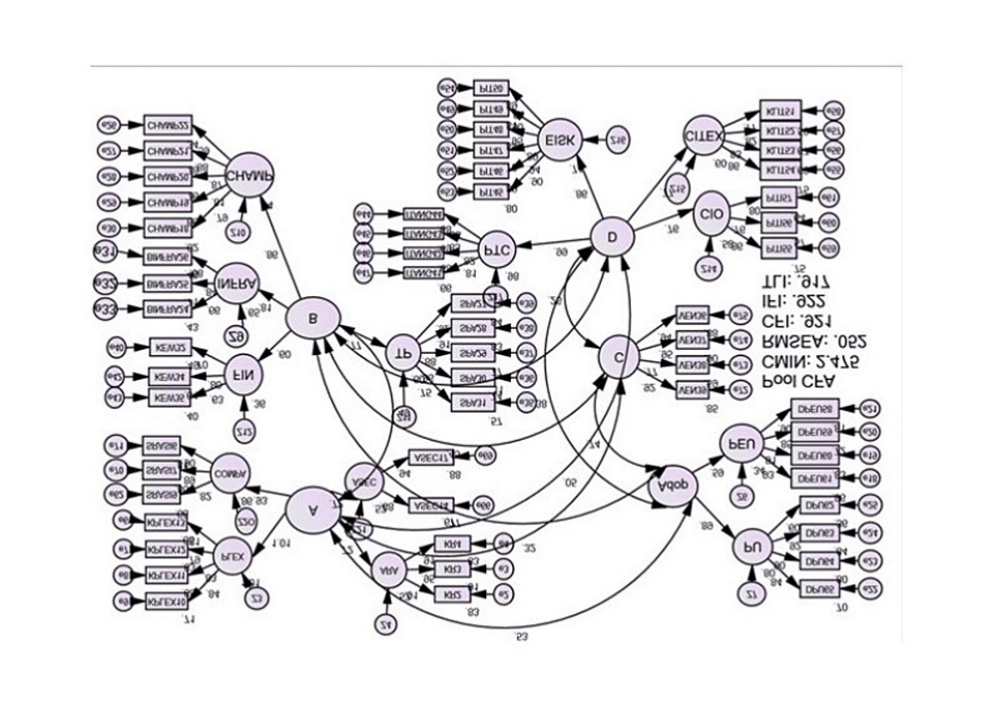
Pooled confirmatory factor analysis of the measurement model.

The second-level constructs were tested separately to match the model desired before testing the entire model (pooled CFA). A combined CFA is also necessary to assess the correlation between all constructs measured in the discriminant validity procedure, where high correlation values above 0.85 are considered to have a shadow over each other. After all requirements for validity and reliability tests were met, structural equation modeling (SEM) was performed, including all items for each construct for the analysis of the standard regression coefficient (standardized regression weight) and the normal regression coefficient (regression weight). Figure 4 displays the SEM analysis of the study. The standardized regression weight values were as follows: technological construct at 0.20, organizational construct at 0.87, environmental construct at 0.46, and human construct at 0.23. An increase of one standard deviation in the technological, organizational, environmental, and human constructs will increase compliance by 0.20, 0.87, 0.46, and 0.23 standard deviations, respectively.

## Discussion

Several statistics were examined to evaluate the psychometric properties, which included: (1) summary statistics of the initial dimension/subscales, (2) initial misfit items statistics (Table 1), (3) category structure (Table 2), (4) psychometric properties of multiple modification rounds (Table 3), and (5) summary statistics of the modified subscales.

Out of the initial 65 items in the questionnaire, items DPTC40, CVEN39, ASEC14, and CVEN38 were identified as outliers and deleted during the first screening. Subsequently, items ACOMPATIBLE8, ACOMPATIBLE9, ACOMPLEX10, ASEC14, ASEC15, BTP31, DEISK46, DEISK47, DEISK48, DCITEX51, DCIO56, and DCIO57, which had negative PMC values, were also deleted as they did not measure the intended parameters. Items DEISK47, ARA1, ACOMPATIBLE9, and DPEU61 were deleted due to factor loadings > 0.7. In total, 18 items were deleted, leaving 49 items for subsequent actual data analysis. Items ARA2 and ARA3 were rephrased.

A 1-4-point Likert scale was sufficient to evaluate the theoretical framework's intention and analyze the questionnaire hypothesis. This was demonstrated by the scale falling within the 1.4 to 5 range [17,26]. The average (in ascending order from negative to positive) also demonstrated that respondents were able to assign value based on the respective items [26]. Figure 2 depicts the clear definition of the scale by the respondents and the absence of continuous numbers in the bell shape [26].

Unidimensionality was demonstrated as all questionnaire items were aligned in the same direction and queried the same value. Furthermore, it also indicated that the questionnaire did not contain more than one dimension, which was evidenced by the 40.5% of the raw variance explained by measures.

The questionnaire demonstrated very good reliability regardless of the presence of internal consistency (Cronbach’s alpha KR20 = 0.93), where the item and person reliability were 0.97 and 0.90, respectively. These values exceeded 0.90, indicating high reliability and substantial acceptability [13,14]. The high person reliability showed that the respondents in this pilot study exhibited varying abilities to respond to the questionnaire, which also related to the Wright map findings [22]. The Cronbach’s alpha (KR20) of 0.93 indicated good internal consistency and qualification for subsequent actual data collection, and the high item reliability proved that the items could be located accurately according to the latent variable [17].

In this study, up to 50% of the respondents agreed that the TPC-OHCIS could ease their daily jobs during the COVID-19 pandemic, while the other half disagreed. Rasch analysis using differential item functioning revealed that nurses were among the disagreeing respondents, as shown in Figure 3.

Advantage of this study

Nurses are the HCWs who use the TPC-OHCIS system most frequently in Malaysian primary healthcare services. Yet, their perception of the system has never been evaluated. Using a validated TPC-OHCIS Questionnaire aids in evaluating the implementation of the TPC-OHCIS application for improvement and national rollout. The appropriate measuring tool used among the implementers of the system will facilitate planning for quality improvement efforts in service delivery.

Limitations of the study

The present study has its limitations, including the use of a convenience sampling method amid the COVID-19 pandemic. This study was conducted in 2021 when movement control orders were implemented in Malaysia. The study was conducted entirely online, and all surveys were distributed to the respondents' superiors without a proper briefing on the overall idea of the research, except for the study information stated at the beginning of the survey Google Form before the respondent consented to participate. There is a possibility of respondent bias, as no supervision or explanation was provided to guide answering the questionnaire, which was self-administered. Therefore, each respondent answered as they perceived fit without understanding the true intent of the survey fully. In addition, the prolonged period of the COVID-19 pandemic may have caused fatigue among health personnel, likely leading them to answer the questions carelessly and without focus. Respondents may have responded to the statements by either agreeing or disagreeing just to complete the form (acquiescence bias).

Recommendation

Future researchers can use more effective methods to avoid sampling bias, such as simple random sampling, which provides equal odds for every member of the population to be chosen as a respondent in the study. We aim to use this validated questionnaire for a national study to cover all states that have installed and implemented the TPC-OHCIS in Malaysia; therefore, verification and improvement of the questionnaire will be undertaken once the actual study is completed.

## Conclusions

Achievement in incorporating four theories in the development of a 65-item questionnaire covering four important scales demonstrated the robustness of the TPC-OHCIS in measuring the primary objective. The validation and reliability assessment, customized to the application and services used, contribute to accuracy in measurement. This tool can assist the Ministry of Health, which owns the TPC-OHCIS application, in evaluating the implementation of its application and in supporting their healthcare providers' services during its implementation. Focusing on the MCH unit, which is a pillar of primary healthcare services, has allowed for a broad scope of implementation stage coverage. Collecting feedback from targeted respondents who used the system enabled identification of the desired characteristics that needed evaluation. Finally, the newly designed TPC-OHCIS questionnaire can be applicable for evaluating other system applications at the primary health clinic level or hospital level, especially when the implementation process requires a collective effort from multiple disciplines or job categories.

## Appendices

Appendix 1

Teleprimary Care Oral Health Clinical Information System (TPC-OHCIS)

(Borang Soal Selidik Sistem Jagaan Tele Primer, Informasi Klinikal Kesihatan Dental (TPC-OHCIS))

Instruction: Please mark (√) in the appropriate Likert scale box.

Scale 1 = Disagree the most, Scale 2 = Disagree, Scale 3 = Agree, Scale 4 = Agree the most

Arahan: Sila tandakan (√) pada kotak skala Likert yang berkenaan.

Skala 1 = Paling tidak setuju, Skala 2 = Tidak setuju, Skala 3 = Setuju, Skala 4 = Paling setuju

**Table 7:** Teleprimary Care Oral Health Clinical Information System (TPC-OHCIS) questionnaire. There are 15 items (10, 11, 12, 13, 33, 39, 40, 41, 42, 48, 49, 50, 53, 54, and 58) that are negative. The Likert scores obtained for these items should be reversed for scoring analysis.

Item		Likert Scale			
		1	2	3	4
Technology scale					
ARA	Relative advantage Kelebihan relatif				
1	Using the TPC-OHCIS application enables me to do my work quickly. Kerja saya lebih cepat selesai dengan menggunakan aplikasi TPC-OHCIS				
2	Using the TPC-OHCIS application improves my quality of work. Kerja saya lebih berkualiti dengan menggunakan aplikasi TPC-OHCIS.				
3	Using the TPC-OHCIS application enhances my job effectiveness. Aplikasi TPC-OHCIS meningkatkan keberkesanan kerja saya.				
4.	Using the TPC-OHCIS application increases my productivity. Aplikasi TPC-OHCIS meningkatkan produktiviti saya.				
5.	Using the TPC-OHCIS application makes my job easier. Kerja saya lebih mudah dengan menggunakan aplikasi TPC-OHCIS.				
ACOM	Compatibility Keserasian				
6.	TPC-OHCIS application can be easily accessed across multiple platforms (laboratory results, X-Ray, and other related patient data). Aplikasi TPC-OHCIS lebih mudah untuk akses pelbagai platform (keputusan makmal, X-Ray dan lain-lain data pesakit)				
7.	TPC-OHCIS application user interfaces provide transparent access to all platforms. Halaman depan aplikasi TPC-OHCIS menyediakan akses untuk memasukkan data terus kesemua platform.				
8.	Data received from other devices (tablet /laptop/smartphone) outside health facilities in the TPC-OHCIS application can be easily merged into the database for analysis. Data yang dimasukkan melalui telefon/laptop ke dalam applikasi TPC-OHCIS boleh disatukan dalam database untuk analisa				
9.	Information data is shared seamlessly across our organization regardless of the location. Data informasi dikongsi tanpa batasan akses dalam organisasi dimana jua.				
ACOMPLEX	Complexity Kerumitan				
10.	I do not know enough about the TPC-OHCIS application to handle my job satisfactorily. Saya susah nak faham penggunaan aplikasi TPC-OHCIS untuk menjalankan kerja dengan baik.				
11.	I need a long time to understand and familiar with the TPC-OHCIS application. Saya ambil masa yang lama untuk pandai menggunakan aplikasi TPC-OHCIS.				
12.	I do not find enough time to study and upgrade my technology skills before using the TPC-OHCIS application. Saya tak ada masa yang cukup untuk belajar dan mahir sebelum menggunakan aplikasi TPC-OHCIS.				
13.	I often find it too complex to understand and use this TPC-OHCIS application. Saya merasakan aplikasi TPC-OHCIS terlalu rumit dan kompleks.				
ASEC	Security concern Isu Keselamatan				
14.	I feel secure in using the TPC-OHCIS application, keying patients’ data, and sharing it across my organization. Saya yakin keselamatan data pesakit yang dimasukkan di aplikasi TPC-OHCIS dan dikongsi dalam organisasi saya.				
15.	I would feel safe using the TPC-OHCIS application to retrieve patient data. Saya rasa data pesakit selamat untuk di muat turun dari aplikasi TPC-OHCIS.				
16.	I am concerned about data patient leakage. Saya prihatin kejadian kecurian data pesakit.				
17.	I am concerned about how much I can trust the vendor. Saya berhati hati dalam memberi sepenuh kepercayaan kepada vendor.				
Organization scale					
BCHAMP	Presence of specified liaison officer Pegawai penyelaras.				
18.	A specified liaison officer will inform top managers and vendors about the faulty TPC-OHCIS application. Pegawai penyelaras akan memberikan maklumbalas kepada Pembekal dan pengurusan tertinggi berkaitan masalah aplikasi TPC-OHCIS.				
19.	A specified liaison officer plays a role in upgrading the TPC-OHCIS application for users’ needs. Pegawai penyelaras memainkan peranan penting bagi menambahbaik fungsi aplikasi TPC-OHCIS.				
20.	A specified liaison officer has a good relationship with both vendors and top managers. Pegawai penyelaras mempunyai hubungan yang baik dengan pembekal dan pengurusan tertinggi.				
21.	A specified liaison officer can train staff to use the TPC-OHCIS application well. Pegawai penyelaras berkebolehan untuk melatih staf menggunakan aplikasi TPC-OHCIS.				
22.	A specified liaison officer provides training/courses for the users a few times a year. Pegawai penyelaras menyediakan latihan /kursus berkaitan sistem beberapa kali setahun.				
BINFRA	Infrastructure Infrastruktur				
23.	We have enough computers for the TPC-OHCIS application use. Kami mempunyai komputer yang mencukupi untuk menggunakan aplikasi TPC-OHCIS.				
24.	We have a reliable computer network in our use. Kami mempunyai jaringan rangkaian komputer yang stabil.				
25.	Appropriate hardware, software, and network infrastructures were in place before the TPC-OHCIS application implementation. Semua keperluan komputer, perisian sistem, rangkaian jaringan telah lengkap sebelum aplikasi TPC-OHCIS diimplementasikan.				
26	Presence of integrated Information System applications encompassing different functional areas (retrieve Lab results, X-Ray, Pharmacy). Terdapat sistem applikasi integrasi informasi merangkumi pelbagai keperluan fungsian (contoh mendapatkan keputusan makmal, X-Ray, Farmasi).				
BTP	Top Management Support Sokongan pegawai atasan.				
27.	Top Management always supports and encourages using the TPC-OHCIS application for job-related tasks. Pegawai atasan sentiasa menyokong penggunaan aplikasi TPC-OHCIS untuk aktiviti kerja berkaitan.				
28.	Top management provides the necessary help and resources to enable staff to use the TPC-OHCIS application. Pegawai atasan menyediakan keperluan dan sumber bagi pekerjanya menggunakan aplikasi TPC-OHCIS.				
29.	Top management provides good access to hardware when staff need it. Pegawai atasan memberikan akses yang baik kepada pekerjanya yang memerlukan perkakasan komputer berkaitan.				
30.	Top management gives feedback to vendors on every dismay or unsatisfactory comment from staff Pegawai atasan memberikan maklumbalas dari stafnya berkaitan masalah sistem kepada pembekal.				
31.	Top management provides good access to the TPC-OHCIS application when staff needs it. Pegawai atasan menyediakan kemudahan internet yang baik kepada stafnya semasa menggunakan aplikasi TPC-OHCIS.				
BFIN	Financial resources Sumber kewangan				
32.	The organization provides sufficient financial aid to coordinate the implementation of TPC-OHCIS Organisasi menyalurkan peruntukan yang mencukupi untuk tujuan menyelaras implementasi TPC-OHCIS.				
33.	I find it difficult to use the TPC-OHCIS application because it cannot be upgraded due to insufficient budget. Saya rasa susah menggunakan aplikasi TPC-OHCIS kerana ianya tidak dapat dinaiktaraf atas masalah peruntukan kewangan.				
34.	Enough computers are available to access the TPC-OHCIS application. Komputer mencukupi untuk menggunakan aplikasi TPC-OHCIS.				
35.	We easily get obsolete computer replacements. Kami mudah mendapatkan penggantian komputer yang telah rosak.				
Environment scale					
CVEN	Vendor support Sokongan dari pembekal				
36.	The vendor entertains each one of our complaints dutifully. Pembekal menangani setiap aduan dengan bertanggungjawab.				
37.	The vendor can upgrade the TPC-OHCIS application according to our needs. Pembekal boleh menaiktaraf aplikasi TPC-OHCIS mengikut kesesuaian/keperluan kerja kami.				
38.	The system vendor attended the technical meetings quite frequently. Pembekal kerap masuk mesyuarat teknikal bersama..				
39.	I have a platform to voice problems regarding the TPC-OHCIS application directly to the vendors. Saya mempunyai platform untuk mengutarakan keluhan berkaitan aplikasi TPC-OHCIS terus kepada pembekal.				
Human resource scale					
DPT	Staff competency in Information technology System Kompetensi staf terhadap sistem informasi teknologi.				
40.	I don’t know how to use a computer. Saya tak pandai guna komputer.				
41.	I never used to work online. Saya tidak pernah melakukan tugasan secara atas talian.				
42.	I need people’s help to use a computer. Saya perlu bantuan orang lain untuk menggunakan komputer.				
43.	I like to work using the online system Saya suka kerja mengunakan sistem atas talian.				
44.	The TPC-OHCIS application is easy to operate. Aplikasi TPC-OHCIS mudah dikendalikan.				
DEIS	Knowledge of the TPC-OHCIS application Pengetahuan mengenai aplikasi TPC-OHCIS				
45.	I have enough training/courses before working with the TPC-OHCIS application. Latihan/kursus yang diberikan mencukupi sebelum saya mula menggunakan aplikasi TPC-OHCIS.				
46.	It took me only a few days to master the TPC-OHCIS application well. Saya hanya mengambil masa beberapa hari sahaja untuk mahir mengendalikan aplikasi TPC-OHCIS.				
47.	The TPC-OHCIS application facilitates task management. Aplikasi TPC-OHCIS memudahkan pengurusan kerja.				
48.	The TPC-OHCIS application is hard to use. Aplikasi TPC-OHCIS susah dikendalikan				
49	I have to open many interfaces to key in one patient’s data. Saya terpaksa membuka banyak interface/ muka untuk menyelesaikan satu kes pesakit.				
50	TPC-OHCIS application takes time because I have to open so many interfaces. Aplikasi TPC-OHCIS mengambil masa lama sebab terpaksa membuka banyak interface/muka.				
DCIT	Clinical information technology competency Kepakaran dalam informasi teknologi klinikal				
51	I have confidence in my ability to operate the TPC-OHCIS application. Saya yakin dengan kemampuan saya mengendalikan aplikasi TPC-OHCIS.				
52	I have the expertise regarding Information technology to provide valuable knowledge to the organization Saya mempunyai kemahiran berkaitan teknologi maklumat yang mungkin berguna kepada organisasi.				
53	It doesn’t make any difference whether I add/ share knowledge with others related to the usage of the TPC-OHCIS application. Tiada perbezaanpun akan berlaku samada saya berkongsi/ menambah pemahaman berkaitan penggunaan aplikasi TPC-OHCIS				
54	I feel that other employees can provide more valuable knowledge about the application’s use. Orang lain boleh memberikan pendidikan yang lebih berguna berkaitan penggunaan aplikasi.				
DCIO	Perceived Innovativeness of Information Technology Officer (ITO) Persepsi Inovatif Pegawai Teknologi Maklumat (PTM).				
55	The ITO is actively considering the introduction of new technology to solve to organization’s problem. PTM sentiasa memperkenalkan fungsi baru bagi membantu menyelesaikan masalah yang dihadapi oleh organisasi.				
56	The ITO tries to keep a technological leading edge by adopting new technology early. PTM sentiasa memperkenalkan sistem baru supaya sentiasa menggunakan teknologi terkini.				
57	The ITO tends to take risks in the decision-making of new technology introduction. PTM cenderung mengambil risiko dalam membuat keputusan memperkenalkan teknologi baru.				
DPEU	Perceived Ease Use Persepsi penggunaan mudah				
58	I often become confused every time I use TPC-OHCIS application. Saya seringkali keliru bila menggunakan aplikasi TPC-OHCIS.				
59	Interacting with the TPC-OHCIS application is frequently frustrating. Mengendalikan aplikasi TPC-OHCIS seringkali membuatkan saya kecewa.				
60	I find the TPC-OHCIS application makes my job easier. Aplikasi TPC-OHCIS memudahkan kerja saya.				
61	The TPC-OHCIS application provides useful guidance in performing tasks. Aplikasi TPC-OHCIS memberikan bimbingan berguna dalam menjalankan tugasan.				
DPU	Perceived Usefulness Persepsi Kebergunaan				
62	My job would be hard to perform without the TPC-OHCIS application. Kerja saya lebih susah kalau tak ada aplikasi TPC-OHCIS.				
63	Using the TPC-OHCIS application improves my job performance Dengan menggunakan aplikasi TPC-OHCIS meningkatkan prestasi kerja saya.				
64	Using the TPC-OHCIS application saves me job time. Dengan menggunakan aplikasi TPC-OHCIS, saya dapat menjimatkan masa kerja.				
65	Using the TPC-OHCIS application supports critical aspects of my job (e.g. retrieving patients with missed treatment). Aplikasi TPC-OHCIS membantu menyelesaikan kerja-kerja sukar saya (contoh mencari pesakit yang cicir rawatan).				

Suggestion for analysis

There was a 65-item TPC-OHCIS questionnaire with four Likert scales (1=highly disagree, 2=disagree, 3=agree, 4=highly agree). To calculate the mean total sum score of each of the four scales (Technology, Organization, Environmental, Human Resource) and the mean total score for each of the 13 subscales:

Technology

·         Relative advantage (5 items)

·         Compatibility(4 items)

·         Complexity(4 items)

·         Security concern (4 items)

Organization

·         Presence of specified liaison officer (5 items)

·         Infrastructure(4 items)

·         Top Management Support(5 items)

·         Financial resources(4 items)

Environmental:

·         Vendor support (4 items)

Human Resource

·         Staff competency in Information technology Systems (12 items)

·         Knowledge of the TPCOHCIS TPCOHCIS (6 items)

·         Clinical information technology competency(4 items)

·         Perceived Innovative Information Technology Officer (ITO)(3 items)

·         Perceived Ease Use (4 items)

·         Perceived Usefulness (4 items)
